# *Machaerium hirtum* (Vell.) Stellfeld Alleviates Acute Pain and Inflammation: Potential Mechanisms of Action

**DOI:** 10.3390/biom10040590

**Published:** 2020-04-11

**Authors:** Juliana Agostinho Lopes, Vinícius Peixoto Rodrigues, Marcelo Marucci Pereira Tangerina, Lucia Regina Machado da Rocha, Catarine Massucato Nishijima, Vania Vasti Alfieri Nunes, Luiz Fernando Rolim de Almeida, Wagner Vilegas, Adair Roberto Soares dos Santos, Miriam Sannomiya, Clélia Akiko Hiruma-Lima

**Affiliations:** 1 Department of Structural and Functional Biology (Physiology), São Paulo State University (UNESP), Institute of Biosciences, Botucatu 18618-970, São Paulo, Brazil; julianaalopes@hotmail.com (J.A.L.); peixoto.rodrigues@unesp.br (V.P.R.); lucia.rocha@unesp.br (L.R.M.d.R.); vaniaalfierinunes@gmail.com (V.V.A.N.); 2 Coastal Campus, São Paulo State University (UNESP), São Vicente 11330-900, São Paulo, Brazil; marcelomptang@hotmail.com (M.M.P.T.); wagner.vilegas@unesp.br (W.V.); 3 Department of Structural and Functional Biology, Institute of Biology, State University of Campinas (UNICAMP), Campinas 13083-862, São Paulo, Brazil; catarinenishijima@gmail.com; 4 Botany Department, São Paulo State University (UNESP), Biosciences Institute, Botucatu 18618-970, São Paulo, Brazil; luiz.rolim@unesp.br; 5 Laboratory of Neurobiology of Pain and Inflammation, Department of Physiological Sciences, Central of Biological Sciences, Federal University of Santa Catarina, Trindade, Florianopolis 88040-900, Santa Catarina, Brazil; adair.santos@ufsc.br; 6 School of Arts, Sciences and Humanities, São Paulo University (USP), São Paulo 03828-000, Brazil; miriamsan@usp.br

**Keywords:** *Machaerium hirtum*, antinociceptive effect, anti-inflammatory action

## Abstract

*Machaerium hirtum* (Vell.) Stellfeld (Fabaceae) known in Brazil as “jacaranda de espinho” or “espinheira santa nativa” is a medicinal plant commonly used in folk medicine to treat ulcers, cough and diarrhea. This study aimed to investigate the anti-inflammatory and antinociceptive effects of hydroalcoholic extracts from *M. hirtum* twig (HEMh) using in vivo experimental models of nociception through the involvement of transient receptor potential channels, acid-sensing ion channel (ASIC), nitrergic, opioidergic, glutamatergic, and supraspinal pathways. Our results revealed an antinociceptive effect of HEMh mediated by the opioidergic, l-arginine-nitric oxide and glutamate systems, as well as by interactions with TRPA1/ASIC channels. The anti-inflammatory effect of HEMh evaluated with a xylene-induced ear edema and by the involvement of arachidonic acid and prostaglandin E2 (PGE_2_) showed involvement of the COX pathway, based on observed decreases in PGE_2_ levels. A phytochemical investigation of the HEMh led to the isolation of α-amyrin, β-amyrin, allantoin, apigenin-7-methoxy-6-*C*-β-d-glucopyranoside, and apigenin-6-*C*-β-d-glucopyranosyl-8-*C*-β-d-xylopyranoside. In conclusion, the acute oral administration of HEMh inhibits the nociceptive behavioral response in animals through the nitrergic, opioid, glutamatergic pathways, and by inhibition of the TRPA1 and ASIC channels, without causing locomotor dysfunction. In addition, its anti-inflammatory effect is associated with the COX pathway and decreased PGE_2_ levels.

## 1. Introduction

The species *Machaerium hirtum* (Vell.) Stellfeld (Fabaceae), popularly known as “barreiro”, “bico-de-andorinha”, “jacarandá bico de pato”, “jacarandá de espinho”, and “espinheira santa nativa”, is a medicinal plant found in Paraná, Mato Grosso, Mato Grosso do Sul, São Paulo, and Minas Gerais in Brazil [1,2,3]. Besides Brazil, this medicinal plant is also found at the borders of Bolivia and Paraguay [3]. The bark of this plant is popularly used to treat ulcers [2], cough [4], diarrhea, and cancer [5]. Phytochemical data from *M. hirtum* leaves included the identification of flavanones, alkaloids, triterpenes, steroids [1], and *C*-glycosylated flavones [4]. Despite the folk medicinal uses of this plant, few pharmacological assays have been conducted on it. The hydroethanolic extract of *M. hirtum* leaves exhibited no mutagenic effects in in vitro assays both on HepG2 cells and on *S. typhimurium* strains (TA97a, TA98, TA100, and TA102), the same study also highlights the chemopreventive potential of this medicinal plant [4]. The crude extract from leaves and twigs of *M. hirtum* and their fractions elicit anti-inflammatory responses, and all samples were able to cause reduction in ear edema and myeloperoxidase (MPO) activity in mice [1]. The therapeutic properties attributed to *M. hirtum* and the demonstration of its anti-inflammatory effects in a previous preliminary study show that there is a need for further studies to confirm and expand this knowledge, as well as to investigate the mechanism of action of this species as a new therapeutic alternative for the treatment of inflammation and pain. Thus, this study aimed to confirm the anti-inflammatory effects of hydroalcoholic extract of *M. hirtum* twigs (HEMh) and to investigate its possible antinociceptive effects using in vitro experimental model of inflammation and nociception caused by chemical or thermal agents. We also investigated the possible involvement of the opioidergic, l-arginine-nitric oxide, and glutamate systems, as well the interactions with transient receptor potential TRP/ASIC channels in HEMh effects. In addition, we also verified the acute oral toxicity, the chemical composition, and the antiulcer and anti-diarrheal action of this extract.

## 2. Materials and Methods

### 2.1. Drugs and Reagents

The chemicals used are as follows: acetic acid (Labimpex, Diadema, Brazil), formaldehyde (Chemco, Campinas, Brazil), arachidonic acid (AA), capsaicin, carbenoxolone, cinnamaldehyde, dexamethasone, indomethacin, l-arginine, N(G)-Nitro-l-arginine methyl ester (L-NAME), l-glutamic acid hydrochloride, menthol, naloxone (Sigma-Aldrich, St. Louis, MO, USA), morphine (Cristália, Itapira, Brazil), lansoprazole (Cruz Vermelha, Botucatu, Brazil), diazepam (Hipolabor, Belo Horizonte, Brazil), piroxicam (Pfizer, São Paulo, Brazil), methanol (MeOH, Sigma-Aldrich, St. Louis, MO, USA), dichloromethane (DCM, Tedia, Rio de Janeiro Brazil), and formic acid (Tedia, Rio de Janeiro, Brazil). A saline solution (0.9% NaCl) was used as vehicle for the drugs, and the solutions were adjusted to pH 7.0 with 3 M NaOH, if necessary.

### 2.2. Collection and Identification of Plant Samples

Samples of twigs from *Machaerium hirtum* (Vell.) Stellfeld were collected at João Hipólito Martins highway (km 3) Botucatu city, São Paulo State, Brazil in December 2012. The botanical taxonomy was identified by Prof. Dr. Luiz Fernando Rolim de Almeida at the Botany Department, Biosciences Institute, São Paulo State University (UNESP), Botucatu, São Paulo, Brazil. A voucher specimen (BOTU 027643) was deposited at the Herbarium BOTU of the same institution. The data collected was recorded in the SisGen platform (National System of Management of Genetic Heritage and Associated Traditional Knowledge) as genetic patrimony under the registration number A1BB684.

### 2.3. Phytochemical Experimental Procedures and Standards

#### 2.3.1. Extraction and Isolation

The air-dried twigs of *M. hirtum* (500 g) were powdered and extraction was performed with 1.5 L 30% (*w*/*v*) hydroethanolic mixture; the resulting suspension was protected from light and percolated at 20 drops/minute. Filtration and evaporation in vacuo resulted in a black residue.

Thin layer chromatographic (TLC) analyses were performed on 200 μm silica gel (Sorbent Technologies^®^, Norcross GA, USA) and visualized using UV light (254 and 365 nm). NMR analyses and 2D experiments were run on Varian^®^ INOVA 500, operating at 500 MHz for ^1^H and 125 MHz for ^13^C (11.7 T), using TMS as an internal standard. Standards of the *C*-flavones apigenin-7-methoxy-6-*C*-β-d-glucopyranoside (**4**) and apigenin-6-*C*-β-d-glucopyranosyl-8-*C*-β-d-xylopyranoside (**5**) were previously obtained from the leaves of *M. hirtum* [6]. The quinic acid and sucrose used as standards during the ESI-MS analysis were from the collection of the Phytochemistry Laboratory of School of Arts, Science and Humanities, São Paulo University, Brazil.

#### 2.3.2. Chromatographic Methods and HPLC-PAD Analysis

TLC analyses were performed on 0.25 mm thick silica gel 60G (Merck, 7731), prepared on glass plates. A mixture of chloroform/methanol/*n*-propanol/water (5:6:1:4, *v*/*v*) was used as an eluent; after solvent evaporation the plates were sprayed with an ethanolic solution of anisaldehyde, sulfuric acid and acetic acid (90 mL: 5 mL: 1 mL), followed by heating. After spraying, the plate was examined under UV light at 254 and 366 nm. Analytical HPLC analysis was performed using an HPLC Jasco^®^ system equipped with 2 pumps, model PU-2086 *Plus*, a dynamic mixer MX-2080-32 and a photodiode array detector MD 2018 *Plus* with a Phenomenex^®^ Synergi hydro RP 18 column (150 × 4.6 mm, 4 μm). A binary gradient elution system with solvent A (0.1% Formic Acid in H_2_O) and solvent B (0.1% Formic Acid in Acetonitrile (ACN) was applied with an initial isocratic elution in a ratio of 85:15 (A:B) for 5 min, followed by a linear gradient formation initially at 85:15 (A:B) to 60% B in 15 min. The flow rate was 1.0 mL/min.

#### 2.3.3. Direct-Injection ESI/MS and ESI/MS/MS Analysis

ESI analysis was carried out on LCQ FLEET Thermo Scientific^®^ equipped with an ion trap analyzer system: data were acquired using Xcalibur 2.1.0.2.40 software. The source temperature was set at 250 °C, and the source voltage was constant at 3.5 kV. Nitrogen was used as a sheath and nebulizer gas at 5 L/min, 10 psi. Helium was introduced into the system at an estimated pressure of 6 × 10^−6^ mbar to improve trapping efficiency and was provided as the collision gas during the CID experiment. For MS/MS spectra, the fragmentation amplitude varied between 0.6 and 0.9 V; the MS operated in the negative ion mode with a scan rate of 13,000 u/s. Samples were infused into the ESI source by use of a syringe pump at a flow-rate of 5 μL/min.

### 2.4. Animals

Adult male and female Swiss mice (25–35 g; approximately 6 weeks of age) and male Wistar rats (160–200 g; approximately 8 weeks of age) were obtained from the Anilab Laboratory Animal Creation and Trade Ltd. (Paulínia, São Paulo, Brazil). All animals were housed collectively in cages and were kept in a controlled environment (22 ± 2 °C, with a 12 h light/dark cycle, lights on at 06:00) with access to water and food (Presence^®^, Brazil) ad libitum. Animals were acclimatized to housing conditions for at least seven days before experiments and all experiments were performed during the light phase of the light/dark cycle. The standard drugs and HEMh were always administered orally (by gavage) using a saline solution (10 mL/kg) as the vehicle. All experiments conducted were in accordance with the Brazilian legislation regulated by the National Council for the Control of Animal Experimentation (CONCEA) and ethical principles in animal research formulated by the Brazilian Society of Science in Laboratory Animals. Animal protocols were approved by the Biosciences Institute/UNESP Ethics Committee on Use of Animals (Approval no 367-CEUA). The number of animals (*n* = 6 to 11 per group) used and the intensity of the noxious stimuli were the minimum necessary to obtain reliable data.

### 2.5. Acute Toxicity Analysis and Hippocratic Screening

Male and female Swiss mice were divided into four groups (*n* = 10 per group), according to sex and treatment, and received either saline solution (10 mL/kg by oral route) or HEMh (5000 mg/kg body weight, p.o.). After treatment, the acute toxicity and behavioral parameters (Hippocratic screening) were analyzed as described by Souza-Brito (1994) [7] and Malone and Robichaud (1962) [8]. Observations were performed at 30, 60, 120, 240, and 360 min after oral treatments. The behavioral parameters, body weights (g), and number of deaths were monitored daily for 14 days. On the 15th day, the animals were killed, and the hearts, lungs, livers, spleens, kidneys, testicles, ovaries, and uteruses were collected, weighed, and subjected to macroscopic analyses. We compared all parameters obtained from mice treated with HEMh with those obtained from the respective control groups that received the vehicle (saline).

### 2.6. Anti-Inflammatory Activity

#### 2.6.1. Xylene-Induced Ear Edema

The animals underwent a two-hour fast and were then administered either vehicle (10 mL/kg) or HEMh (62.5, 125, and 250 mg/kg) orally by gavage and intraperitoneally with dexamethasone (5 mg/kg) used as positive control (*n* = 8–10 per group). One hour after vehicle or HEMh treatment and two hours after dexamethasone treatment, 20 μL xylene was applied topically to the inner and outer surfaces of the right ear of the mouse; the left ear was used as a control. One hour after xylene application, the animals were killed and circular sections (7 mm of diameter) of the ears were collected and weighted. Edema was expressed as the weight difference (mg) between the right and left ear measured with an analytical balance [9].

#### 2.6.2. Ear Edema Induced by Arachidonic Acid

The possible effects of HEMh on inflammation caused by arachidonic acid (from porcine liver) was evaluated as previously described by Young et al. (1984) [10] with a few modifications. Mice were fasted for two hours and then administered orally by gavage with vehicle (10 mL/kg) or HEMh (62.5, 125, and 250 mg/kg) and intraperitoneally with dexamethasone (5 mg/kg) used as positive control (*n* = 8–10 per group). One hour after oral treatment and two hours after dexamethasone treatment, 10 μL of arachidonic acid (2 mg/ear) was applied topically to the inner and outer surfaces of the right ear of the mouse; the left ear was used as a control. One hour after arachidonic acid application, the mice were killed and circular sections (7 mm of diameter) of the ears were collected and weighted. Edema was expressed as the weight difference (mg) between the right and left ear, measured with an analytical balance.

In addition to this, to assess the possible action of HEMh in the COX pathway, PGE_2_ concentrations in the right ears of animals treated with vehicle, HEMh, or dexamethasone [11,12] were quantified as described above. To this end, each ear was homogenized in 0.7 mL of 0.1 M phosphate buffered solution (pH = 7.25) containing 1 mM EDTA. The samples were incubated at 0 °C for 15 min and then centrifuged at 12,290× *g* for 15 min at 4 °C. The supernatant was collected and stored at −80 °C until the determination of PGE_2_ levels using enzyme-linked immunosorbent assay/ELISA (KGE004B, R&D Systems, Minneapolis, MN, USA).

### 2.7. Antinociceptive Activity

#### 2.7.1. Formalin-Induced Nociception

The model used was as described by Hunskaar and Hole (1987), with a few modifications [13,14]. Male Swiss mice (*n* = 6−10 per group) were treated by gavage with HEMh (32.25, 62.5, 125, or 250 mg/kg, oral) or vehicle (saline 10 mL/kg, oral), used as negative control. One hour after treatment all mice received an intraplantar injection of 20 µL 2.7% formalin solution (1% formaldehyde) in saline on the ventral surface of the right hind-paw. After formalin injection, the animals were immediately placed into glass cylinders (20 cm) and the time (seconds) spent licking the injected paw was recorded with a chronometer as an indicator of nociception (time of licking). The mice were observed during the first 5 min (neurogenic phase) and between the fifteenth and thirtieth minute (inflammatory phase). The lowest effective dose of HEMh that induced an antinociceptive effect in both phases was used to characterize the mechanism of action.

#### 2.7.2. Hot Plate Test

Thermal hypersensitivity after HEMh administration was evaluated using a hot plate test, as described by Eddy and Leimbach (1953) with modifications [15,16]. Male Swiss mice (*n* = 8–10 animals per group) were treated with HEMh (62.5 mg/kg, by oral route), vehicle (saline, 10 mL/kg, by oral route), or morphine (5 mg/kg, subcutaneous) used as a positive control. One hour after oral and 30 min after the subcutaneous treatments the mice were placed on a heated metal plate (Ugo Basile, Gemonio VA, Italy) with a temperature set at 50 ± 1 °C (sensitizes more C and type II A δ nociceptors) or 56 ± 1 °C (sensitizes type I Aδ nociceptor). The time (in seconds) until the mouse manifested a nociceptive behavior (lifting or licking of the hind-paw) was considered as the latency response to the thermal stimuli. A cut-off time of 20 s was chosen to avoid tissue injury. The animals were pre-selected (24 h before starting the experiment), excluding those with a response time of less than 4 s or greater than 11 s. This latency response was recorded at 60, 90, 120, and 150 min following oral treatment and the prolongation of the latency time (s) compared with the values of the control group (vehicle) was used for statistical comparison.

### 2.8. Analysis of the Possible Mechanisms of Action of HEMh

#### 2.8.1. Locomotor Performance

In order to rule out antinociception false positive results, the effects of HEMh on spontaneous locomotor activity of male Swiss mice (*n* = 8 per group) was tested on the rotarod apparatus (Insight Ltd., Ribeirão Preto, Brazil). This method was used to eliminate any possible non-specific muscle relaxant effects of this extract [14,17]. Twenty-four hours before the experiments, mice capable of remaining on the rotarod (4 cm in diameter, 6 rpm) for three periods of 60 s without falling were pre-selected. On the day of the experiment, the mice were treated with either vehicle (saline, 10 mL/kg, given by oral route), HEMh (62.5 mg/kg, given by oral route), or diazepam—a positive control (2 mg/kg, given by intraperitoneal route). One hour after oral treatment (saline or HEMh) and 30 min after diazepam injection, the mice were placed on the apparatus and the number of falls from the apparatus over 180 s was recorded with a stopwatch.

#### 2.8.2. Involvement of Glutamatergic System, Transient Receptor Potential Cation Channel Subfamily V Member 1, A Member 1, and M Member 8 (TRPV1, TRPA1, and TRPM8, Respectively) and Acid-Sensing Ion Channel (ASIC)

To evaluate the effect of HEMh on the glutamatergic system and on the TRPV1, TRPA1, TRPM8, and ASIC channels, specific activators of each channel were used. Male mice (*n* = 6–10 per group) were pretreated orally with HEMh (62.5 mg/kg) or saline (10 mL/kg), one hour before intraplantar injections of algogenic substances. Then, the mice received 20 µL of glutamic acid (30 µmol/paw, pH = 7), capsaicin (2 µmol/paw), cinnamaldehyde (40 nmol/paw), menthol (2 µmol/paw), or acidified saline (3% acetic acid, pH = 2) into the ventral surface of the right hind paw. Animals were placed individually in a glass cylinder and were observed for 15 min [(glutamic acid), 6 min (capsaicin (TRPV1) and cinnamaldehyde (TRPA1)] and 20 min [menthol (TRPM8) and acidified saline (ASIC)], according to the procedures previously described with modifications. The amount of time, in seconds, spent licking the injected paw was recorded and was considered indicative of nociception [14,18,19,20].

#### 2.8.3. Involvement of the l-Arginine–Nitric Oxide Pathway in Antinociception

To evaluate the involvement of the l-arginine–nitric oxide system in the antinociceptive activity of HEMh, male Swiss mice (*n* = 8–11 animals per group) were pre-treated with l-arginine (500 mg/kg, intraperitoneal) or saline (10 mL/kg, intraperitoneal—control). After 30 min, the mice received either vehicle (10 mL/kg, p.o.), HEMh (62.5 mg/kg, p.o.) or l-NAME (65 mg/kg, by intraperitoneal route), an inhibitor of NO synthesis, used as positive control. The nociceptive response to formalin intraplantar injection was recorded 1 h after the administration of HEMh or vehicle and 30 min after the administration of l-NAME [21,22].

#### 2.8.4. Involvement of the Opioid System in Antinociception

To assess whether the opioid system mediated the antinociceptive effect of HEMh, male Swiss mice (*n* = 6–10 animals per group) were administered naloxone, a non-selective opioid receptor antagonist (1 mg/kg, intraperitoneal) or saline (10 mL/kg, intraperitoneal). After 30 min, the mice received either vehicle (10 mL/kg, p.o.), HEMh (62.5 mg/kg, p.o.) or morphine (2.5 mg/kg, subcutaneous) used as positive control. The nociceptive response to the formalin intraplantar injection was recorded 1 h after the administration of HEMh or vehicle and 30 min after the administration of morphine [21,22].

### 2.9. Involvement of the Opioid System in Intestinal Transit

To assess whether the opioid system mediated the antipropulsive effect of HEMh, male Swiss mice (*n* = 6–9 animals per group) received pre-treatment with naloxone, a non-selective opioid receptor antagonist (15 mg/kg, intraperitoneal) or saline (10 mL/kg, intraperitoneal). After 1 h of pre-treatment, mice were orally administered with a suspension of 10% activated charcoal (10 mL/kg, p.o.), after 30 min the mice were killed, and the distance traveled by the charcoal and the total size of the small intestine were calculated [23].

### 2.10. Evaluation of Antiulcer Activity in the Gastric Ulcer Induced by Ethanol or Indomethacin (NSAIDs)

Male Wistar rats (*n* = 7–8 animals per group) were separated into groups and deprived of food for 16 h with water ad libitum. Vehicle (saline 10 mL/kg), carbenoxolone (positive control 100 mg/kg against gastric ulcer induced by ethanol), lansoprazole (positive control 30 mg/kg against gastric ulcer induced by indomethacin), or HEMh (62.5, 125 or 250 mg/kg) were administered orally. One hour later, gastric lesions were induced by administration with 1 mL absolute ethanol [24] or 30 min later, gastric lesions were induced by indomethacin (50 mg/kg, solubilized in sodium carbonate 0.5%, pH 7.4) [25]. All animals were killed 1 h after absolute ethanol or 6 h after oral administration of indomethacin. The stomachs were removed and opened along the greater curvature, and lesion area (mm^2^) was determined using the program AVSoft BioView^®^ (Campinas, São Paulo, Brazil).

### 2.11. Statistical Analyses

The results are expressed as mean ± standard errors of the mean (S.E.M.) of the parameters obtained. Parameters were analyzed using one-way ANOVA followed by Dunnett’s or Tukey’s post hoc tests to compare three or more groups or by Student’s *t*-test to compare two groups. In all analyses, *p* values were considered statistically significant only if they were less than 0.05. The statistical software GraphPad Prism^®^ v6 (San Diego, CA, USA) was used for calculation.

## 3. Results

### 3.1. Phytochemical Profile of HEMh

The mixture of water and 70% ethanol can be considered a suitable solvent to obtain large quantities of this material (79.1 g). The TLC analysis indicated the presence of flavonoids, sugars, and triterpenes. The yield of this extract was 15.8% *w*/*w* of the powdered plant. The extract was dissolved in a mixture of *n*-butanol/water (1:1, *v*/*v*) and after separation, the phases were evaporated in a vacuum to obtain butanolic and aqueous fractions (2.1 g and 2.4 g, respectively). The butanolic fraction (2.1 g) was fractionated by gel permeation column chromatography (CC). The column was packed with Sephadex (LH-20, 57 cm × 3.0 cm i.d.) and soaked in a ratio of Methanol: Water (8:2, *v*/*v*). The column was then eluted with the same solvent mixture, yielding 115 fractions. After TLC analysis, similar fractions were combined to yield 10 subfractions. Subfraction 2 (120 mg), after being submitted to CC fractionation using a mixture of solvents hexane/chloroform 90:10 (*v*/*v*), resulted in a white solid (6.0 mg, compounds **1** and **2**). Part of subfraction 6 (78 mg) was fractionated by CC using a mixture of chloroform/methanol/water 80:18:2 (*v*/*v*) as a solvent. Subfractions 4–8 yielded a yellow solid (4.0 mg, compounds **3** and **4**). Subfraction 10 was eluted on a PVPP (polyvinylpolypyrrolidone) column with methanol as solvent to give 5 (14 mg).

**Figure 1:**
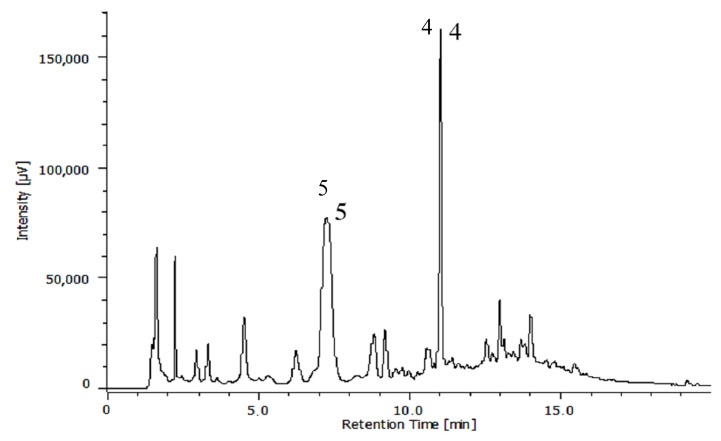
HPLC-PAD analysis of hydroalcoholic extract from the *M. hirtum*.

Phytochemical studies of the HEMh allowed the isolation of five compounds. Compounds **1** and **2** were identified as α-amyrin (**1**) and β-amyrin (**2**) based on their ^13^C NMR spectra and comparison with the literature data [26]. The identification of allantoin (**3**) was permitted after the analysis of ^13^C NMR and ^1^H NMR spectral data [27]. Compound **3** was reported for the first time in the *Machaerium* genus. Compounds **4** and **5** were identified by comparison of their spectral data with authentic standards, which defined the structures as apigenin-7-methoxy-6-*C*-β-d-glucopyranoside (**4**) and apigenin-6-*C*-β-d-glucopyranosyl-8-C-β-d-xylopyranoside (**5**), respectively. The chromatographic profile of HEMh was determined by HPLC-PAD as shown in Figure 1. The comparison of the retention times and the UV spectrum with authentic standards of the peaks with retention times of 7.2 and 11.1 min (Figure 1) indicates that they correspond to apigenin-7-methoxy-6-*C*-β-d-glucopyranoside (**4**) and apigenin-6-*C*-β-d-glucopyranosyl-8-*C*-β-d-xylopyranoside (**5**), respectively.

**Figure 2:**
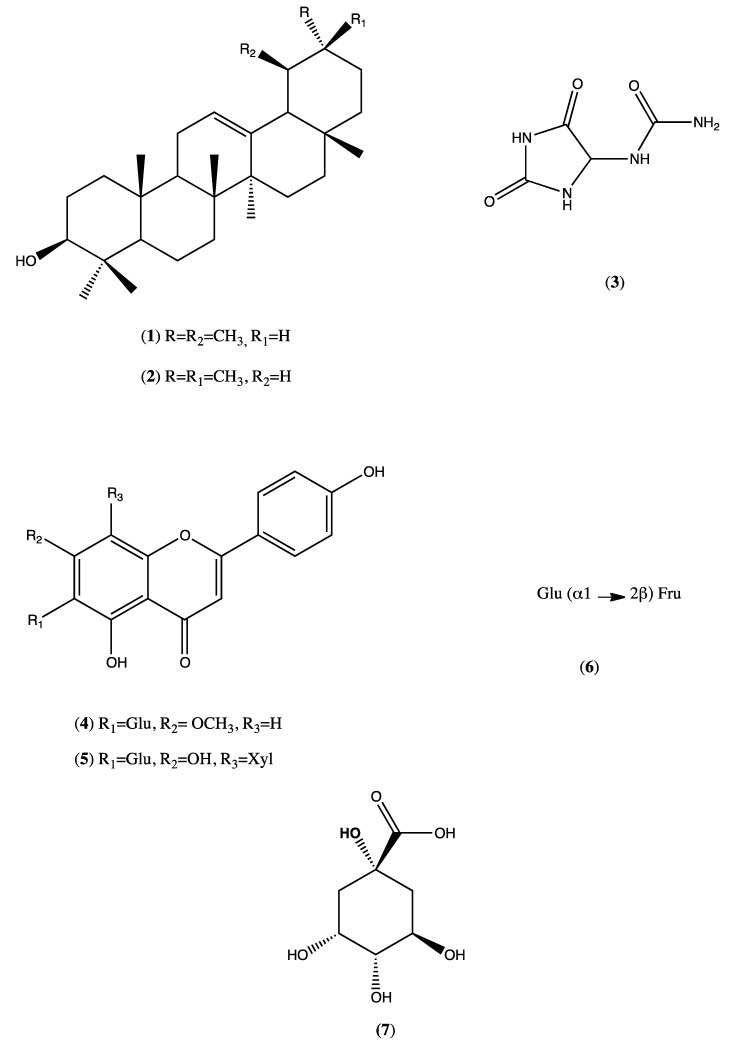
Compounds isolated and identified from *Machaerium hirtum*.

The dissolution of the HEMh in methanol and subsequent ESI/MS analysis in a full scan range (0 to 500 *m*/*z*) showed that the extract contained a mixture of compounds, including apigenin-7-methoxy-6-*C*-β-d-glucopyranoside (**4**), apigenin-6-*C*-β-d-glucopyranosyl-8-*C*-β-d-xylopyranoside (**5**), sucrose (**6**), α-amyrin (**1**), β-amyrin (**2**), and quinic acid (**7**) (Figure 2). Under the conditions described above, the negative ion mode ESI/MS spectrum obtained (see Figure S1 in Supplementary Materials) showed three deprotonated molecules [M − H] at *m*/*z* 341, 445, and 563, related to the molecular weights of *C*-flavones [28,29].

Information regarding the fragmentation of each deprotonated molecule was obtained from direct-injection ESI/MS using an ion trap analyzer. Table 1 shows the important fragmentation patterns of the precursor ions of the HEMh obtained by direct injection ESI/MS/MS analysis together with data for the standard compounds analyzed under the same conditions. The results showed the characteristic fragment ions and relative abundances of the precursor ions at *m*/z** 190, 341, 445, 563 of the extract exactly matched those of the authentic standards 7, 6, 4, and 5, respectively. Moreover, the ESI/MS/MS fragmentation data were in full agreement with the proposed identities. The direct-injection ESI/MS/MS analyses confirmed the presence of apigenin-7-methoxy-6-*C*-β-d-glucopyranoside (**4**), apigenin-6-*C*-β-d-glucopyranosyl-8-*C*-β-d-xylopyranoside (**5**), which were isolated by phytochemical analysis, while sucrose (**6**), and quinic acid (**7**) were only detected in the HEMh. MS^n^ fragmentation of the precursor ion [M − H]^−^ at *m*/*z* 341 in the ESI negative mode showed the presence of the product fragment ions at *m*/*z* 178 and 161 respectively, which characterizes the clear losses of hexose and pentose unities, confirming the presence of sucrose 6 in this extract [30].

In the case of apigenin-7-methoxy-6-*C*-β-d-glucopyranoside (**4**), apigenin-6-*C*-β-d-glucopyranosyl-8-*C*-β-d-xylopyranoside (**5**), ESI-MS analysis showed the presence of ions of [*M* − H − 60]−, [*M* − H − 90]− and [*M* − H − 120]−, which were demonstrated by Becchi and Fraisse (1989) [31] to be characteristic ions of *C*-flavonoids. The methoxylated flavone apigenin-7-methoxy-6-*C*-β-d-glucopyranoside (**4**) exhibited a significant [*M* − H − CH_3_]^−•^ radical anion base peak, but no other fragmentation in the MS^2^ spectra has been observed (Table 1).

### 3.2. Acute Toxicity and Hippocratic Screening

**Figure 3:**
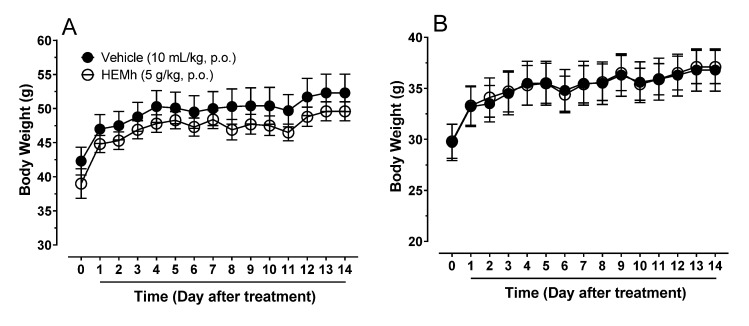
Body weight changes in male (**A**) and female (**B**) mice after acute oral administration (5000 mg/kg) of hydroalcoholic extract of the twigs of *Machaerium hirtum* (HEMh). The results are expressed as the mean of the values obtained in 10 animals. Student’s *t*-test. *p* > 0.05.

As part of the pharmacological evaluation, HEMh was first investigated for acute toxicity in male and female Swiss mice using Hippocratic screening. We did not observe any behavioral changes in the male or female Swiss mice throughout the observation period (data not shown). In the fourteen days following the administration of a single oral dose of HEMh (5000 mg/kg, p.o.) none of the treated mice of either sex produced any visible signs or symptoms of toxicity. No animals died, and no significant changes in organ weights or daily body weights were observed after acute oral treatment with HEMh (Table 2 and Figure 3).

### 3.3. The Anti-Inflammatory Activity of HEMh on Ear Edema Induced by Xylene or Arachidonic Acid and PGE_2_ Quantification

We characterized the anti-inflammatory activity of HEMh (by oral route), using the xylene-induced ear edema model. As shown in Table 3, the weight of the mouse ear significantly increased due to edema caused by xylene application. The positive control dexamethasone inhibited edema by 70% (*p* < 0.001). Treatment with HEMh caused a significant reduction in edema at doses of 125 and 250 mg/kg with a reduction of 39% (*p* < 0.05) and 47% (*p* < 0.01) respectively, when compared to the control group treated with the vehicle.

Based on the anti-inflammatory activity of HEMh in mice ear edema, we investigated the mechanisms related to this anti-inflammatory action using arachidonic acid (AA). In this model, animals pretreated orally with all doses of HEMh showed a decrease in edema with reductions of 26–40% when compared to the control group treated with vehicle (Table 3). The positive control, dexamethasone, inhibited edema by 59% (*p* < 0.001). To assess whether HEMh acts on the COX pathway, the PGE_2_ was quantified on the ears from mice in this model. The result presented in Table 3 shows that all doses of HEMh decrease (33%–52%) the PGE_2_ concentration promoted by AA. However, only oral HEMh pretreatment at doses of 62.5 and 250 mg/kg significantly decreased PGE_2_ levels (*p* < 0.05) compared to the vehicle-treated control group (Table 3). The positive control dexamethasone also decreases PGE_2_ levels by 65% (*p* < 0.001).

### 3.4. Effect of HEMh on Formalin-Induced Nociception

**Figure 4:**
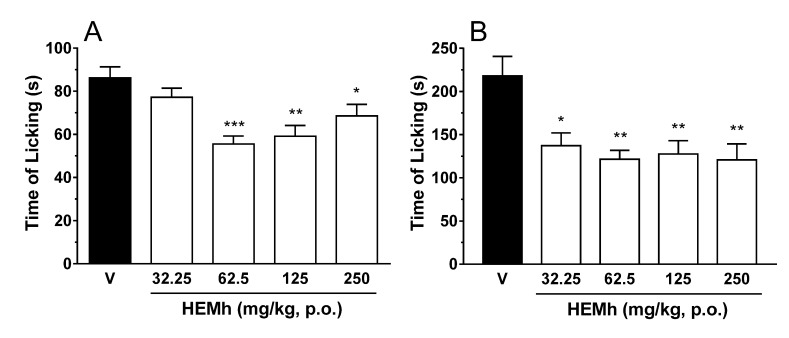
Effect of different doses of hydroalcoholic extract of the twigs of *Machaerium hirtum* (HEMh) in the formalin test (panel **A**—neurogenic phase and panel **B**—inflammatory phase). Each column represents the mean obtained from the 6–10 animals in each group (V = vehicle and different doses of HEMh), while the vertical lines indicate the S.E.M. The asterisks denote the significance levels compared with the control group. * *p* < 0.05, ** *p* < 0.01 and *** *p* < 0.001 calculated using one-way ANOVA (analysis of variance) followed by Dunnett’s test.

The results depicted in Figure 4 (panel A and B) show that oral treatment with HEMh administered at different doses of 62.5, 125 and 250 mg/kg caused a significant antinociceptive effect in both phases (neurogenic and inflammatory) of the formalin test. Levels of inhibition compared to the control group were 35%, 32%, and 20% in the neurogenic phase, and 44%, 42%, and 45% in the inflammatory phase, respectively. However, the 32.25 mg/kg dose of HEMh was effective only in the second phase (inflammatory pain) of formalin nociception. According to these results, we chose an effective dose of HEMh (62.5 mg/kg) to further investigate its antinociceptive effects.

### 3.5. Hot Plate Test

The results depicted in Table 4 show that treatment with HEMh (62.5 mg/kg) did not affect the latency in the heat-induced nociceptive response at a temperature of 50 ± 1 °C (sensitizes more C and type II Aδ nociceptors) nor 56 ± 1 °C (sensitizes type I Aδ nociceptor) throughout the observation period (*p* > 0.05). Under similar conditions, morphine-treated mice (5 mg/kg) showed a significant and marked latency increase (*p* < 0.05) in the hot plate assay for both temperatures throughout the observation period, when compared to control animals.

### 3.6. Effect of HEMh on Locomotor Performance

**Figure 5:**
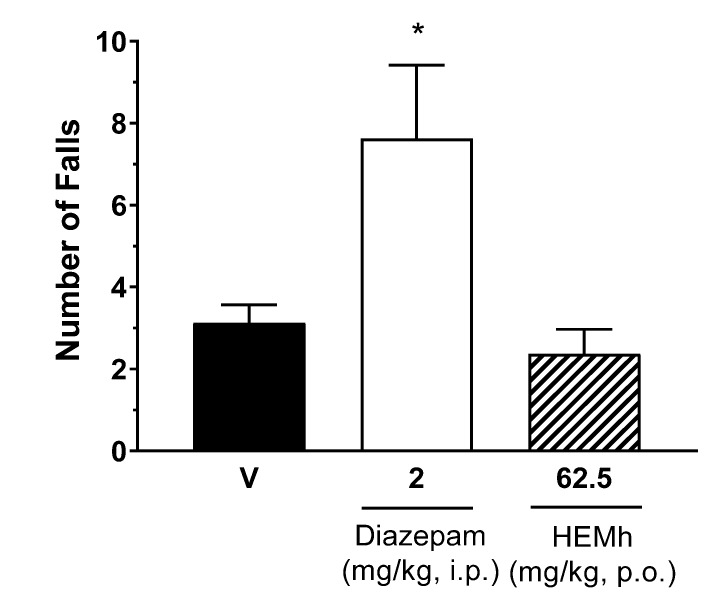
Effect of the hydroalcoholic extract of the twigs of *Machaerium hirtum* (HEMh) and diazepam (control positive) on the locomotor performance of mice in a rotarod test. The results are the number of falls in each group of animals expressed as the mean of values obtained in 8 animals per group and the vertical lines indicate the S.E.M. Statistical analyses were performed using one-way ANOVA (analysis of variance) followed by Tukey´s post hoc test (vehicle (V) vs. Diazepam * *p* < 0.05).

The motor performance of mice was not significantly affected by the oral administration of HEMh at a dose of 62.5 mg/kg (*p* > 0.05) compared to vehicle control group. Only treatment with diazepam (2 mg/kg) significantly decreased (*p* < 0.05) the endurance time on the rotating rod and increase the number of falls when compared with the time of the control group treated with vehicle (Figure 5).

### 3.7. Involvement of the Glutamatergic System, TRPV1, TRPA1, TRPM8, and ASIC Channels in HEMh Actions

**Figure 6:**
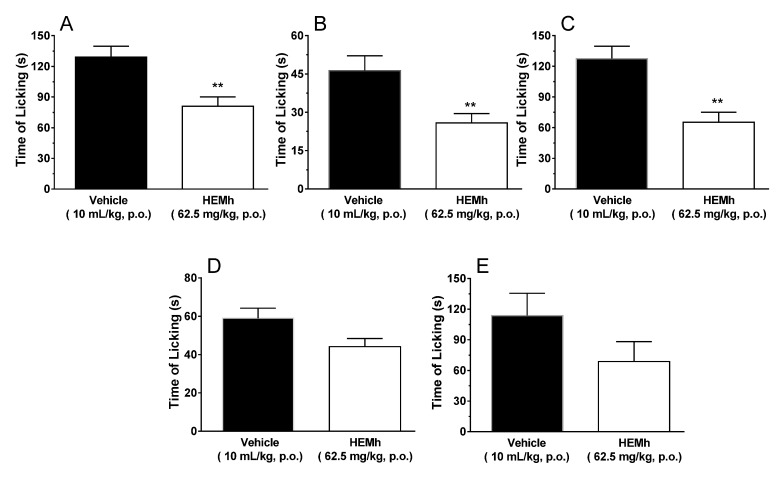
Effects of the hydroalcoholic extract of the twigs of *Machaerium hirtum* (HEMh) on the nocifensive behavior of mice induced by an intraplantar injection of glutamate (**A**), cinnamaldehyde (**B**), acidified saline (**C**), capsaicin (**D**), and menthol (**E**). The column represents the mean of 6–10 animals and the vertical lines indicates the S.E.M. The asterisks denote the significance levels compared with the vehicle control group (** *p* < 0.01) using Student’s *t*-test.

The results depicted in Figure 6 show that HEMh was able to significantly reduce nociception caused by glutamate (inhibition of 37%, *p* < 0.01), cinnamaldehyde (inhibition of 44%, *p* < 0.01) and acidified saline (inhibition of 49%, *p* < 0.01) compared to the control group, indicating that HEMh can act directly or indirectly on the glutamatergic system and TRPA1 and ASIC channels, respectively (Figure 6A–C). However, HEMh at a dose of 62.5 mg/kg did not cause statistically significant reduction in nociception caused by capsaicin nor menthol when compared to the control group (*p* > 0.05), suggesting that both channels do not contribute to the antinociceptive effect of the extract (Figure 6D,E).

### 3.8. Involvement of the l-Arginine–Nitric Oxide Pathway in HEMh Action

**Figure 7:**
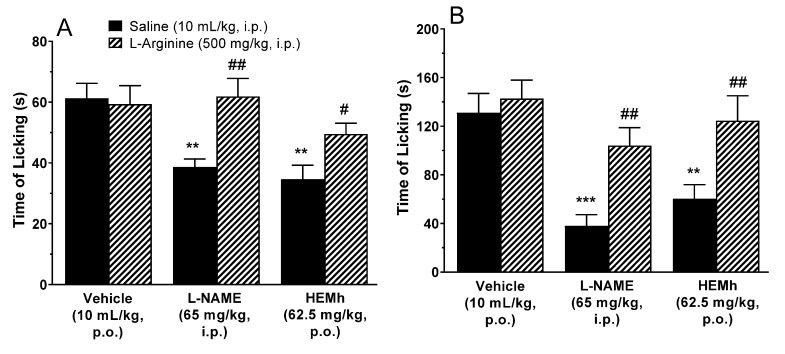
Involvement of the l-arginine-nitric oxide pathway in the antinociceptive activity of hydroalcoholic extract of the twigs of *Machaerium hirtum* (HEMh) in the formalin test (panel **A**—neurogenic phase and panel **B**—inflammatory phase). Each column represents the mean of 8–11 animals and the vertical lines indicate the S.E.M. The asterisks denote the significance levels compared with the vehicle control group (** *p* < 0.01 and *** *p* < 0.001) and a hashtag denotes the significance levels when comparing the l-NAME and HEMh treatments with their respective saline group (^#^ *p* < 0.05, ^##^ *p* < 0.01) using one-way ANOVA (analysis of variance) followed by Tukey’s test.

To investigate the possible role of nitric oxide (NO) in the antinociceptive mechanism of action of this extract, we used l-arginine as a substrate for NO synthesis. The results presented in Figure 7 show that 30 min prior treatment of animals with the NO precursor l-arginine (500 mg/kg, i.p.) completely reversed the antinociceptive response caused by HEMh (62.5 mg/kg, p.o.) and L-NAME (65 mg/kg, i.p., used as positive control), when analyzed against both the neurogenic and inflammatory phases of formalin-induced licking (Figure 7A,B).

### 3.9. Involvement of the Opioid System in HEMh Actions

**Figure 8:**
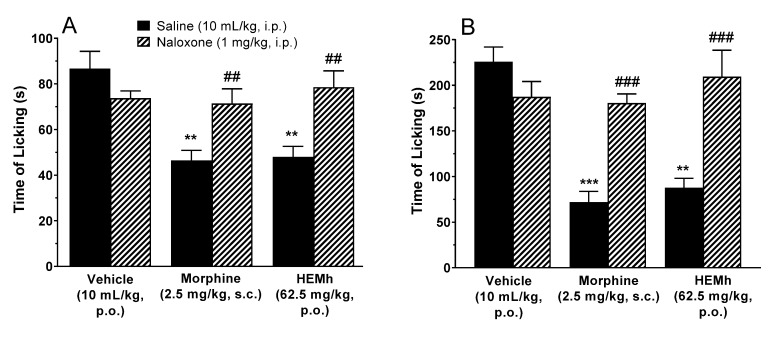
Involvement of the opioid system on antinociceptive activity of hydroalcoholic extract of the twigs of *Machaerium hirtum* (HEMh) in the formalin test (panel **A**—neurogenic phase and panel **B**—inflammatory phase). Each column represents the mean of 6–10 animals and the vertical lines indicates the S.E.M. The asterisks denote the significance levels compared with the vehicle control group (** *p* < 0.01 and *** *p* < 0.001) and hashtag denotes the significance levels when comparing the morphine and HEMh treatments with their respective saline group (^##^ *p* < 0.01, ^###^ *p* < 0.001) using one-way ANOVA (analysis of variance) followed by Tukey’s post hoc test.

The results in Figure 8 show that the pretreatment of animals with naloxone (non-selective antagonist of the opioid receptor) completely reversed the antinociceptive effect caused by morphine in the neurogenic (*p* < 0.01) and inflammatory phases (*p* < 0.001). Under the same conditions, naloxone also significantly antagonized the antinociceptive action of HEMh in both phases (*p* < 0.01) confirming that the opioid system also contributes to the antinociceptive effect of this extract.

Besides the antinociceptive effects of the opioid system, the efficacy of opiates is well established as an anti-diarrheal agent. Our results (Table 5) show that the group of animals treated with HEMh inhibited the intestinal propulsion (22%) in relation to the control group (*p* < 0.05). Under the same conditions, morphine promoted a large reduction in intestinal propulsion (44%) in relation to the control group (*p* > 0.001). Our results also show that the pretreatment of animals with naloxone (non-selective antagonist of opioid receptor) completely reverses the inhibitory effect of intestinal propulsion caused by HEMh and morphine (*p* < 0.01). Both results (Figure 8 and Table 5) confirms involvement of the opioid system in HEMh action.

### 3.10. Evaluation of Antiulcer Activity of HEMh in Gastric Ulcers Induced by Ethanol and Indomethacin

The results in Table 6 show that only the highest dose of HEMh (250 mg/kg) significantly inhibited ethanol-induced ulcer by 66% (*p* < 0.0001) and also inhibited indomethacin-induced ulcer by 45% in rats (*p* < 0.05) when compared to the control group treated with vehicle. It is important to note that none of the doses of the extract worsens the gastric lesion induced by injurious agents (*p* > 0.05). The positive control groups (carbenoxolone and lansoprazole) inhibited gastric lesions by 89% and 95%, respectively (*p* < 0.0001).

## 4. Discussion

The phytochemical investigation of the HEMh using chromatographic isolation and/or HPLC/PAD as well as ESI/MS/MS analyses led to the identification of α-amyrin, β-amyrin, allantoin, apigenin-7-methoxy-6-*C*-β-d-glucopyranoside, apigenin-6-*C*-β-d-glucopyranosyl-8-*C*-β-d-xylopyranoside, sucrose and quinic acid. Ignoato et al., (2013) [1] also investigated the crude extract of leaves and branches of *M. hirtum* and between these two studies, only two compounds (flavones) were common. Almost all of the seven compounds isolated from *M. hirtum* exhibit anti-inflammatory and/or antinociceptive actions, thus indicating the great pharmacological potential of this species [32,33,34,35].

As predicted in studies developed by Ignoato et al. (2013) [1], which show anti-inflammatory activity from the hydroethanolic extract of *M. hirtum* in rodents, in the present study, we further investigated the pharmacological action of this extract. Here we examined the antinociceptive effects of HEMh and tried to unravel the mechanisms of action involved in mediating this effect. We demonstrated for the first time that acute oral administration of HEMh causes potent inhibition of the nociceptive behavioral response in animal models of pain through the nitrergic, opioid, and glutamatergic pathways, as well as by inhibition of the TRPA1 and ASIC channels, without causing locomotor dysfunction. In addition to the antinociceptive effect, this study extended the investigation of the anti-inflammatory effect of *M. hirtum*, demonstrating that this action is associated with the COX pathway, based on the observed decreases in PGE_2_ levels without causing gastric lesions, a common side effect of NSAIDs.

Considering the pharmacological potential of medicinal plants in general, it would be useless to conduct a study ignoring the risks of toxicity. Therefore, the first step in this pharmacological study was to ensure the safety of the HEMh. The acute toxicity model combined with a Hippocratic screening aim to obtain information on toxicity and behavioral parameters that may be indicative of adverse effects [36]. This model was chosen to evaluate the acute oral toxicity of HEMh, as it also allowed us to evaluate the effects of toxic metabolites of this extract, which could be detected by monitoring the body weight of animals over the subsequent 14 days [7]. The absence of toxicity signs of the extract motivated us to continue the pharmacological evaluation of HEMh.

The formalin test is an important method for the evaluation of the antinociceptive and analgesics effects of a medicinal plant and their mechanisms of action. This in vivo experimental method resembles pain clinical models in the response to continuous pain associated with tissue injury [37]. This test can be divided into two phases (early and late phases). The early phase (neurogenic phase) is characterized by neurogenic pain, mediated by activation of the transient receptor potential of transient ankyrin calcium channels (TRPA1) and release of glutamate, substance P and calcitonin gene-related peptide (CGRP), inducing hyperalgesia through intracellular messengers and dorsal root ganglion sensitization [38,39]. The late phase of formalin-induced nociception (inflammatory phase) is characterized by the release of various proinflammatory agents, due to tissue damage caused by formalin, which alter the cellular environment, interfering with the process of nociception [40]. In addition, it has been demonstrated that the intraplantar injection of formalin in rodents increases spinal levels of excitatory amino acids, PGE_2_, NO, tachykinin, kinins, among other peptides [21,22,37]. We selected 62.5 mg/kg as the effective dose for subsequent experiments as it shows antinociceptive effects during both phases. Also, the antinociception caused by HEMh was unlikely to be secondary to its non-specific muscle relaxant effect or its specific and/or non-specific depressant central effects, as revealed by the lack of important motor dysfunction or detectable side effects in the rotarod locomotor performance test [41]. Our results demonstrated the ability of HEMh to inhibit nociception in both phases of the formalin test, indicating a possible involvement of the opioid, nitric oxide, glutamate, TRPs, and ASIC channels in mediating the analgesic/anti-inflammatory effect of this extract. The presence of apigenin-7-methoxy-6-*C*-β-d-glucopyranoside and apigenin-6-*C*-β-d-glucopyranosyl-8-*C*-β-d-xylopyranoside in the HEMh phytochemical profile could explain the antinociceptive effect. A previous study by Pinheiro et al., (2012) [42] demonstrated the antinociceptive effect of isolated apigenin, which reduced the licking response in both phases of the formalin model, similar to HEMh.

The mechanisms of action underlying the antinociceptive effects of HEMh were evaluated by studying the involvement of the supraspinatus response in nociception [41,43], through the hot plate test using two temperature ranges. The temperature set at 50 ± 1 °C sensitizes the nociceptors C and type II A δ while 56 ± 1 °C sensitizes nociceptor A I type I [44]. Our results demonstrated that pretreatment with HEMh did not increase the response latency in any of the observation periods. This result ruling out the involvement of the supraspinal response in the nociception of the extract.

NO is a small gas molecule which can be produced from l-arginine by the enzyme NO-synthase, which plays important roles in peripheral and central synaptic transmissions, as well as in the development and maintenance of pain [45]. In the formalin test l-arginine enhances NO synthesis [46] which modulates the excitability of both pre- and post-synaptic neurons in the first synapse of the ascending pathway via action on NMDA receptors [47,48]. We undertook to elucidate the mechanism by which HEMh exerted its antinociceptive activity over the NO pathway. The results showed a significant reversal of the HEMh effect, indicating the effectiveness of this method, thus suggesting that the nitrergic system is a potential target of HEMh. There is already a description in the literature the role of quinic acid derivatives (a component of HEMh), in attenuating the LPS-induced pro-inflammatory response in microglial BV2 cells via action of NO, indicating the involvement of constituents of this extract in anti-inflammatory action [35].

Our results also show the involvement of the glutamatergic system in the antinociceptive mechanism of HEMh. Glutamate, in addition to NO, is another target of nociception. Glutamate is an excitatory amino acid largely distributed in the central nervous system, where it participates in several physiological processes through the activation of ionotropic and metabotropic receptors [49]. Ionotropic receptors are permeable to Na^+^, K^+^, and Ca^2+^, whereas metabotropic receptors are coupled to protein G and act indirectly on ionic channels to modulate them [19]. Glutamate intraplantar injection activates the NMDA receptor, resulting in the release of substance P and CGRP in the central terminal of the first order nociceptor; promoting Ca^2+^ influx, prostanoids and NO production in dorsal horn cells [50,51].

It has been established that the nociceptive effect of NO and the release of glutamate and other excitatory amino acids by nociceptors is modulated by the transient receptor potential (TRP) family [52,53]; with that in mind the involvement of ionic channel associated receptors in the antinociceptive effect of HEMh were investigated. TRP receptors are cation-permeable ionic channels related to a myriad of functions, from taste translation to mechanisms of vasomotor control, including nociception [54]. TRPV1, TRPA1, and TRPM8 are expressed in nociceptors where they detect noxious thermal, chemical and mechanical stimuli; each receptor is activated by distinct sources of stimuli, TRPV1 is activated by noxious heat and capsaicin, TRPA1 is activated by several chemical agents such as alicin, isothiocyanates, and cinnamaldehyde, while TRPM8 is sensitive to cold and menthol [55,56]. We observed that HEMh did not reverse nociception caused by capsaicin (an activator of the TRPV1 channel) or menthol (an activator of the TRPM8 channel) when compared to the control group, thus disproving the involvement of TRPV1 and TRPM8 in the nociception activity of the extract. ASICs belong to the degenerin/epithelial sodium channel (DEG/ENaC) family; they are permeable to cations and play an important role in inflammatory nociception, as these channels are activated by extracellular H^+^, whose concentration become elevated during inflammatory situations [57,58]. Our results indicated the involvement of TRPA1 and ASIC channels in the antinociceptive mechanism of HEMh.

It has been established that TRPA1 directly mediates neurogenic nociception through release of substance P and CGRP [59]; furthermore, it is known that TRP receptors modulate the release of NO and glutamate, both of which are factors related to HEMh action. In addition to involvement of TRPA1, NO, and glutamate, another important modulator of nociception and pain is the opioid system; its receptors are widely distributed in several tissues, including the dorsal horn of the spinal cord [60], where endogenous or exogenous opioids activate these receptors, inhibiting the release of neurotransmitters in the presynaptic fibers and hyperpolarizing the postsynaptic fiber [61]. Our results show that administration of opioid antagonist significantly antagonized the antinociceptive action of HEMh revealing the involvement of the opioid system in the effect of HEMh.

Pinheiro et al., (2012) [42] demonstrated that the antinociceptive effect of apigenin, present in HEMh, seem to involve the participation of the opioid system when it was reversed by naloxone. Besides apigenin, the presence of α- and β-amyrin, two phytochemical compounds from HEMh, could also be related to the antinociceptive action of this extract, if it can be demonstrated that they are involved with the opioid receptor. Lima-Júnior et al., (2006) [62] suggest that the effect of both compounds could be used to treat visceral intestinal pain. Besides these, allantoin could also be responsible for the antinociceptive effect of HEMh. Florentino et al., (2016) [33] attributed a peripheral antinociceptive action related to the opioid receptor to allantoin.

The involvement of the opioid system with HEMh action was also indicated by the HEMh-mediated inhibition of intestinal transit. Among the compounds present in HEMh, emphasis should be placed on allantoin. Zhang et al. (2019) [63] described the effect of allantoin on the accelerated recovery from acute diarrhea, indicating that our results regarding the inhibition of the intestinal propulsion induced by the opiodergic receptor was potentiated by the antidiarrheal action of this medicinal plant. The results presented in this study further confirm the other ethnopharmacological action attributed to this medicinal plant: Its efficacy in combatting diarrhea [5].

Besides the antinociceptive action of HEMh and considering the results of the extract on the inflammatory phase in the formalin model, the focus of this study was also to assess the anti-inflammatory action of this extract. The xylene-induced ear edema model is a reliable test for screening anti-inflammatory natural products [64]. Xylene induces the release of neuropeptides, such as substance P and CGRP, causing vasodilatation and edema [65,66,67]. The arachidonic acid is a precursor of important mediators of inflammation such as prostaglandin E_2_ and LTB4, when applied topically this acid cause a short but intense inflammatory response [68,69] which was used to determine if the extract had any effect on the arachidonic acid pathway. HEMh was capable of reducing edema induced by arachidonic acid, but this effect was only related to the cyclooxygenase pathway, since animals treated with HEMh presented lower PGE_2_ levels than the vehicle-treated group. These results are corroborated by the data from Shoubaky et al., (2016) [70] which demonstrated the anti-inflammatory effect of apigenin by reduced levels of PGE_2_. Besides apigenin, Otuki et al. (2005) [32] evaluated the mixture of alpha-amyrin and beta-amyrin and demonstrated that this mixture produced consistent peripheral, spinal, and supraspinal antinociception in rodents and the mechanisms involved in their action seem to involve the inhibition of protein kinase A- and protein kinase C-sensitive pathways. α,β-amyrin exhibits long-lasting antinociceptive and anti-inflammatory properties via activation of cannabinoid receptors and by inhibiting the production of cytokines and cyclooxygenase 2 [71]. In addition to its antinociceptive properties, β-amyrin also exhibits anti-inflammatory effects, including reduction of inflammation in microglial cells [72,73]. All these data corroborate the characterization of the phytochemical composition and pharmacological (anti-inflammatory and antinociceptive) action of this extract.

Gastric ulceration is a major limitation of the prolonged use of anti-inflammatory drugs for inflammation and chronic pain [74]. We observed the protective effects of HEMh on gastric ulceration induced by ethanol and indomethacin. Our results showed that HEMh did not cause the development of gastric injury, but in high doses, this extract was able to promote gastroprotection. The gastroprotective action of this extract can be attributed to the presence of allantoin, which has already been proven to have an antiulcerogenic effect [75]. This result, in addition to the acute toxicity of HEMh, rules out the side effects of this medicinal plant.

## 5. Conclusions

The present study reveals the effect of HEMh action on TRPA1 receptors, associated with its effect on the opioid system, and that it is responsible for a diminished release of glutamate and NO, which partially explains this extract’s antinociceptive properties. Another important outcome of this study is the anti-inflammatory effect the extract exhibited, which is related to inhibition of cyclooxygenase action, consequently decreasing the production of inflammatory mediators, leading to a lower activation of ASIC receptors. The HEMh effect on the opioid system would also contribute to inflammatory antinociception, the absence of acute toxicity and gastric injury.

## Figures and Tables

**Table 1:** Compounds identified in *Machaerium hirtum* by ESI-MS^n^.

Compounds	Peak No	Deprotonated Ion [M − H]^−^	MS^n^ Ions (*m*/*z*)
**7**	1	191	85, 93, 111, 127, 173
**6**	2	341	161, 179
**4**	3	445	325, 355, 385, 430, 445
**5**	4	563	443, 473, 503, 563

**Table 2:** Toxicological parameters after the acute administration (5000 mg/kg) of hydroalcoholic extract of the twigs of *Machaerium hirtum* (HEMh) in adult Swiss mice through the oral route (*n* = 10).

Sex	Treatment (p.o.)	Dose	Liver	Heart	Lung	Kidney	Spleen	Testicles/Ovaries and Uterus	Deaths
♂	Vehicle	-	12.73 ± 0.27	3.83 ± 0.07	4.20 ± 0.12	6.62 ± 0.10	2.96 ± 0.11	4.06 ± 0.14	0
	HEMh	5000 mg/kg	12.90 ± 0.14	3.80 ± 0.05	4.25 ± 0.05	6.80 ± 0.10	3.32 ± 0.15	4.07 ± 0.06	0
♀	Vehicle	-	12.77 ± 0.34	4.01 ± 0.12	5.29 ± 0.48	6.32 ± 0.19	3.31 ± 0.12	12.76 ± 1.33	0
	HEMh	5000 mg/kg	12.55 ± 0.25	4.03 ± 0.08	4.90 ± 0.25	6.66 ± 0.35	3.40 ± 0.11	8.23 ± 2.73	0

The results are expressed as the mean ± S.E.M. of the relative organ/total body weight of the animals. This ratio was converted into arcsine values for statistical adjustment. The statistical significance was determined by Student’s *t*-test (*p* > 0.05).

**Table 3:** Evaluation of the anti-edematogenic activity of hydroalcoholic extract from the twigs of *Machaerium hirtum* (HEMh) in mice ear edema induced by xylene or arachidonic acid.

Ear Edema Model	Treatment (p.o.)	Dose	Ear Edema (mg)	Inhibition (%)	Prostaglandin E_2_ (pg/tissue)	Inhibition (%)
**Xylene (40 µL)**	Vehicle	-	7.61 ± 0.72	-		
	Dexamethasone	5 mg/kg	2.22 ± 0.41 ***	70		
	HEMh	62.5 mg/kg	6.34 ± 0.85	-		
		125 mg/kg	4.64 ± 0.79 *	39		
		250 mg/kg	4.05 ± 0.57 **	47		
**Arachidonic acid (20 µL)**	Vehicle	-	23.23 ± 1.38	-	1.15 ± 0.19	-
	Dexamethasone	5 mg/kg	9.57 ± 1.10 ***	59	0.41 ± 0.04 **	65
	HEMh	62.5 mg/kg	13.99 ± 1.45 ***	40	0.55 ± 0.12 *	52
		125 mg/kg	17.25 ± 1.22 **	26	0.77 ± 0.15	33
		250 mg/kg	16.19 ± 1.43 **	30	0.61 ± 0.17 *	47

Data represent the mean ± S.E.M. of 8–10 animals per group. The asterisks denote the significance levels compared with the control group treated with vehicle. * *p* < 0.05, ** *p* < 0.001 and *** *p* < 0.0001 using one-way ANOVA followed by Dunnett’s test.

**Table 4:** Effects of hydroalcoholic extract from the twigs of *Machaerium hirtum* (HEMh) on the response latency (s) at 60, 90, 120, and 150 min after the administration of two different temperatures in the hot-plate test in mice.

Temperature	Treatment	Dose	60 min	90 min	120 min	150 min
50 ± 1 °C	Vehicle	-	11.10 ± 0.74	11.10 ± 0.86	12.00 ± 1.23	12.10 ± 0.48
	Morphine	5 mg/kg	23.50 ± 1.66 ***	22.60 ± 1.22 ***	21.10 ± 0.88 ***	19.20 ±1.65 ***
	HEMh	62.5 mg/kg	13.10 ± 1.22	13.22 ± 1.43	14.70 ± 0.84	10.11 ± 0.54
56 ± 1 °C	Vehicle	-	7.25 ± 0.72	6.92 ± 0.48	5.75 ± 0.34	5.24 ± 0.36
	Morphine	5 mg/kg	10.25 ± 0.49 **	9.31 ± 0.88 *	9.87 ± 0.93 ***	9.66 ± 0.90 ***
	HEMh	62.5 mg/kg	6.75 ± 0.72	6.84 ± 0.68	7.00 ± 0.91	6.84 ± 0.50

Data are reported as the mean ± S.E.M. for *n* = 8–10 per group. The asterisks denote the significance levels compared with the control group (vehicle). * *p* < 0.05, ** *p* < 0.001 and *** *p* < 0.0001 using One-way ANOVA followed by Dunnett’s test.

**Table 5:** Effects of hydroalcoholic extract from the twigs of *Machaerium hirtum* (HEMh) on intestinal propulsion in mice with naloxone (opioid receptor antagonist).

Pre-Treatment	Treatment	Dose	Distance Moved by Charcoal (cm)	Inhibition (%)
Saline	Vehicle	-	0.84 ± 0.04	-
Saline	Morphine	2.5 mg/kg	0.48 ± 0.05 ***	44
Saline	HEMh	62.5 mg/kg	0.65 ± 0.04 *	22
Naloxone	Vehicle	-	0.94 ± 0.03	-
Naloxone	Morphine	5 mg/kg	0.82 ± 0.05 ^##^	-
Naloxone	HEMh	62.5 mg/kg	0.82 ± 0.04 ^##^	-

Data are reported as the mean ± S.E.M. of 6–9 animals per group. One-way ANOVA followed by Tukey´s test, * *p* < 0.05, *** *p* < 0.001 represents the difference in relation to the control group treated with vehicle. ^##^ *p* < 0.01 represents the difference in relation to group with different pre-treatments and same treatment.

**Table 6:** Effects of hydroalcoholic extract from the twigs of *Machaerium hirtum* (HEMh) on acute gastric lesion induced by absolute ethanol or NSAIDs in rats.

Gastric Lesion Models	Treatment (p.o.)	Dose (mg/kg)	Lesion Area (mm^2^)	Inhibition (%)
Absolute Ethanol	Vehicle	-	582.70 ± 60.31	-
	Carbenoxolone	100	42.12 ± 18.65 ***	89
	HEMh	62.5	520.52 ± 42.05	-
		125	447.50 ± 69.89	-
		250	197.90 ± 47.80 ***	66
NSAIDs	Vehicle	-	21.96 ± 1.44	-
	Lansoprazole	30	1.03 ± 0.43 ***	95
	HEMh	62.5	19.30 ± 2.72	-
		125	14.98 ± 3.09	-
		250	12.05 ± 1.84 *	45

Data represents the mean ± S.E.M. of 7–8 rats per group. The asterisks denote the significance levels compared with the control group treated with vehicle. * *p* < 0.05 and *** *p* < 0.0001 using One-way ANOVA followed by Dunnett’s test.

## Supplementary Materials

The following are available online at https://www.mdpi.com/2218-273X/10/4/590/s1, Figure S1: The negative direct-injection ESI/MS spectrum of hydroalcoholic extract from the *M. hirtum*.
