# Supplementary material for: Machaerium hirtum (Vell.) Stellfeld Alleviates Acute Pain and Inflammation: Potential Mechanisms of Action
Source: Biomolecules. 2020 Apr 11;10(4):590. doi: 10.3390/biom10040590 (PMC7226113; doi:10.3390/biom10040590)
Supplement: Supplementary file 1 [file biomolecules-10-00590-s001.pdf]

Supplementary material

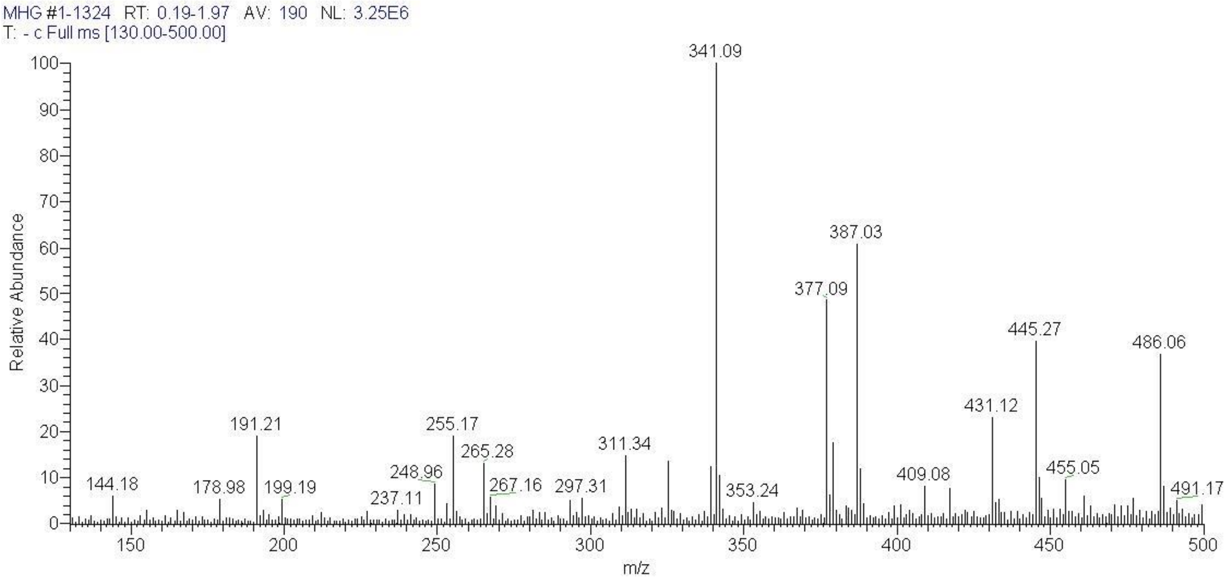

Figure S1. Electrospray ionisation–mass spectrometry (ESI-MS) spectrum of *Machaerium hirtum* twig extract.
